# Illness recognition, decision-making, and care-seeking for maternal and newborn complications: a qualitative study in Jigawa State, Northern Nigeria

**DOI:** 10.1186/s41043-017-0124-y

**Published:** 2017-12-21

**Authors:** Vandana Sharma, Jessica Leight, Fatima AbdulAziz, Nadège Giroux, Martina Bjorkman Nyqvist

**Affiliations:** 1 Abdul Latif Jameel Poverty Action Lab, Massachusetts Institute of Technology, 30 Wadsworth St, Cambridge, MA 02142 USA; 2 Department of Economics, American University, Washington, DC USA; 3 Planned Parenthood Federation of Nigeria, Abuja, Nigeria; 4 Paris School of Economics, Paris, France; 5 Department of Economics, Stockholm School of Economics, Stockholm, Sweden; 6 Present address: Harvard T.H. Chan School of Public Health, 677 Huntington Avenue, Boston, MA 02115 USA

**Keywords:** Maternal mortality, Neonatal mortality, Maternal complications, Newborn complications, Recognition, Care-seeking, Jigawa, Nigeria, Sub-Saharan Africa

## Abstract

**Background:**

Maternal mortality and newborn mortality continue to be major challenges in Nigeria, with the highest levels in the northern part of the country. The objective of this study was to explore the process and sequence of symptom recognition, decision-making, and care-seeking among families experiencing maternal and neonatal illness and deaths in 24 local governmental areas in Jigawa State, Northern Nigeria.

**Methods:**

This qualitative study included 40 illness narratives (ten each for maternal deaths, perceived postpartum hemorrhage (PPH), neonatal deaths, and neonatal illness) that collected data on symptom recognition, perceptions of the causes of disease, decision-making processes, the identity of key decision-makers, and care-seeking barriers and enablers. Data were transcribed verbatim, translated to English, then coded and analyzed using Dedoose software and a codebook developed a priori based on the study’s conceptual model.

**Results:**

Compared to maternal cases, much less care-seeking was reported for newborns, especially in cases that ended in death. Key decision-makers varied by type of case. Husbands played the lead role in maternal death and neonatal illness cases, while female relatives and traditional birth attendants were more involved in decision-making around perceived PPH, and mothers were the principal decision makers in the neonatal death cases. Demand for health services is high, but supply-side challenges including low quality of care, uncertain availability of health workers, and drug stock-outs are persistent. There is a strong belief that outcomes are controlled by God and frequent use of spiritual care sometimes contributes to delays in seeking facility-based care.

**Conclusion:**

These findings suggest key differences in recognition of complications, decision-making processes, and care-seeking patterns between maternal and newborn illness and death cases in Jigawa, Northern Nigeria. Interventions that provide more targeted messaging specific to case and symptom type, are inclusive of family members beyond husbands, and address gaps in quality and availability of care are urgently needed. It may also be important to address the widespread perception that adverse outcomes for mothers and newborns are controlled by fate and cannot be prevented.

## Background

Maternal mortality and newborn mortality are major challenges in Nigeria. Though home to 2% of the world’s population, Nigeria accounts for more than 10% of the world’s maternal and child deaths [1]. The most recent national data estimates Nigeria’s maternal mortality ratio (MMR) at 576 maternal deaths per 100,000 live births (95% CI 500–652) [2]. Neonatal mortality rates (NMRs) at the national level also remain high at 37 deaths per 1000 live births [2]. Both MMR and NMR show a wide geographic variation, with the highest rates in the northern regions. A recent study estimated the MMR in four states in Northern Nigeria (Jigawa, Katsina, Yobe, and Zamfara) to be 1271 per 100,000 live births, corresponding to a lifetime risk of maternal death of 9% [3].

For every maternal death, estimates suggest another 20 women experience pregnancy-related complications [4]. Overall, 15% of women experience maternal complications, but access to emergency obstetric care and delivery with a skilled birth attendant (SBA) can improve survival [4]. Recognition of complications and timely, appropriate care-seeking are thus essential to reduce maternal and newborn mortality [5]. Thaddeus and Maine developed the *three-delay* model, a framework for organizing barriers to recognition and appropriate care-seeking for maternal complications [6]. The categories include (1) delays in deciding to seek care, (2) delays in reaching a health facility, and (3) delays in receiving quality care at the facility.

In Northern Nigeria, many factors contribute to the extremely poor outcomes for mothers and neonates including weak health infrastructure, low literacy, and large distances from health facilities [7]. A shortage of SBAs was partially addressed by the Federal Ministry of Health’s Midwives Service Scheme (MSS), which deployed trained midwives to primary health centers (PHCs) to provide 24-h maternity care [8]. However, skilled birth attendance has remained low. The most recent Demographic and Health Survey reports that in Jigawa State in Northern Nigeria, only 6.7% of women delivered in a health facility and 7.6% of women delivered with a skilled provider [2]. The low use of maternal health services in this context is driven by cultural norms, limited support for accessing maternal health services by husbands, and low knowledge of danger signs and available services [9, 10]. Less is known about recognition and care-seeking for newborn complications in Nigeria. However, in other settings, barriers include poor recognition of symptoms and severity, poor quality of health services, and cost [11].

The objectives of this qualitative study in Jigawa, Northern Nigeria, were to investigate (1) recognition, decision-making, and care-seeking among families who experienced a maternal death, a reported postpartum hemorrhage (PPH), a neonatal death, or an illness within the first 28 days of life; (2) sequences of care-seeking actions; (3) the role of husbands in these processes; and (4) how perceptions of risk influence decision-making.

## Methods

This study was nested within an ongoing cluster randomized controlled trial (RCT) of community-based interventions to reduce maternal mortality in Jigawa State, Northern Nigeria. The trial is being implemented by the Abdul Latif Jameel Poverty Action Lab (J-PAL) and the Planned Parenthood Federation of Nigeria (PPFN) to assess the impact of three interventions: (1) training local women as community resource persons (CoRPs) who provide education and referrals to pregnant women and their families, (2) the CoRPs program plus safe birth kit distribution to pregnant women, and (3) the CoRPs program plus community dramas to change social norms on maternal health.

### Study site

Jigawa State’s population was 4.3 million during the 2006 census [12]. The state is divided into 27 local government areas (LGAs), with 80% of the population living in rural areas [12]. The RCT is being conducted in 96 clusters of villages across 24 LGAs, covering an estimated population of 280,000. LGAs were included if they had a PHC that was part of the MSS. The RCT baseline sample consists of women of reproductive age in a 15% subsample of households randomly selected at baseline between December 2011 and May 2012 (*N* = 7069). A RapidSMS surveillance system, where local women were trained in each village to report vital events using text messaging, was implemented to track births and deaths of women and infants. For all births in baseline households, questionnaires were administered within 3 days after birth and at 28 days after birth to capture data on the pregnancy, delivery, and postpartum period. For deaths of women of reproductive age, verbal autopsies were conducted to determine the cause of death.

### Study design and sampling method

This qualitative study included data on 40 cases, equally divided between four categories (maternal deaths, reported PPH, neonatal deaths, and neonatal illnesses); one to five witnesses were present for each interview (see Table 1). Inclusion criteria for maternal cases included the following: female, aged 18–49 years, gave birth in the previous 6 months, residing in a study village, and family was willing to participate. For maternal death cases, the woman died during pregnancy, childbirth, or within the 42 days after delivery. For PPH cases, the woman reported severe bleeding after childbirth (cases defined as perceived excessive bleeding, rather than clinically diagnosed PPH; further referred to as PPH cases).

**Table 1:** Summary of types of cases and number of interviews

Type of case	Target group	Methodology	No. of interviews
Maternal death	2–3 people present	Illness narrative	10
Perceived postpartum hemorrhage	Women and 2–3 people present	Illness narrative	10
Neonatal death	Woman and 2–3 people present	Illness narrative	10
Neonatal illness	Women and 2–3 people present	Illness narrative	10
Total			40

Inclusion criteria for newborn cases included the following: born in the last 6 months in a study village and family was willing to participate. For newborn death cases, the newborn died within 28 days after birth due to any cause. Neonatal illness cases included newborns experiencing ill-health within the first 28 days after birth. Maternal and newborn cases were included from both the control and the CoRPs arms of the RCT.

Potential cases were identified prospectively and sequentially, using the RapidSMS surveillance system, until the target numbers were reached. Reported PPH, neonatal illness, and neonatal death cases were identified via maternal and newborn complication data from the 3- and 28-day after-birth questionnaires, while maternal death cases included those where the death had been verified and a verbal autopsy was conducted. Those cases that met the study eligibility criteria via the surveillance data were first verified by the field team and then visited. In some cases, decisions to further pursue eligible cases were based on logistics, cost, and geographical considerations. Confirmed cases that were visited and provided informed consent were interviewed. Case finding occurred between June and August 2015.

### Data collection

Illness narratives, qualitative group interviews including the person who experienced the illness, along with several others who were witness to it, were conducted for all cases between June and November 2015. All cases had a sufficient number of witnesses to report on the event. Witnesses included husbands, family members, neighbors, and in a few cases, traditional birth attendants (TBAs). Interview guides were developed based on the conceptual model presented in the accompanying methods paper which concentrates on delays 1 and 2 of the three-delay model and specifically around recognition, decision-making, and care-seeking [13]. The interview guides were translated to Hausa and piloted extensively. They contained open- and close-ended questions on symptom type, severity, and duration; the process to decide on appropriate actions; and care-seeking behavior. All witnesses present during the interviews were given the opportunity to speak. In cases where a witness was particularly quiet, the interviewer encouraged and facilitated their participation in the discussion. In the few situations where there were conflicting viewpoints or contradictory statements, the interviewer probed further and clarified each person’s perspective.

Data were collected by trained interviewers and note-takers (two males and two females) who conducted interviews in pairs in Hausa. These data collectors were recruited from the study areas specifically for this research, and their age and acceptability to the target population were important considerations in the selection process. Interviews were audio recorded and transcribed, and expanded notes were created using notes, memory, and the audio. Illness timelines were constructed and verified to document the sequence of events including symptom recognition, decision-making, and care-seeking steps and the timing of each. Interviews were on average between 20 and 60 min.

Supervisors provided ongoing assistance for quality assurance and periodic re-training. Interviewers completed a debriefing template following each interview including additional notes about the data collection process.

### Analysis

Data were transcribed verbatim by the field team, translated to English, and cross-checked for accuracy by Hausa speakers who compared English transcripts to the audio recordings. A codebook, developed a priori based on the study’s conceptual model (focusing on delays 1 and 2 of the three-delay model), was used as a basis for coding the expanded notes. Dedoose qualitative software (www.dedoose.com) was used for the coding and analysis by two researchers (VS, NG). This software was chosen because its cloud-based platform allows researchers in different countries to work collaboratively in real time in an efficient way.

Coding was done both within cases and across cases and focused on emerging themes related to recognition of illness, decision-making, patterns of care-seeking, as well as barriers and enablers to care-seeking. Thematic content analysis was conducted to understand the processes around recognition and decision-making and the sequence of care-seeking and whether these varied by case type. Data and quotations were summarized in a matrix permitting comparison of the main themes associated with each case separately for recognition, decision-making, and care-seeking. Frequency analysis was conducted to assess factors related to each category. Differences in recognition, decision-making, and care-seeking between the CoRPs intervention arm and the control arm will be explored in a separate publication.

### Ethical approval

Verbal informed consent was obtained from all respondents. Ethical approval was obtained from the Massachusetts Institute of Technology (MIT) and the Jigawa State Operations Research Advisory Committee (ORAC). The trial is registered at clinicaltrials.gov (NCT01487707).

## Results

### Background characteristics

Ten illness narratives for each case type were completed. Most women were between 19 and 29 years, and the majority delivered at home (Table 2). Ten of 20 newborns died during the neonatal period (within 28 days after birth), with seven deaths within 1 day after birth, two between days 1 and 7, and one between days 8 and 28. Cases from 16 LGAs were included.

**Table 2:** Characteristics of women and newborns

Variable	Total, *N* (%)	Maternal death, *N* (%)	Reported PPH, *N* (%)	Neonatal death, *N* (%)	Neonatal illness, *N* (%)
Woman’s age (years)	23.0 (5.66)	23.3 (6.46)	22.6 (4.87)	–	–
Woman’s age (years)					
< 19	3 (15.0)	2 (20.0)	1 (10.0)		
19–29	10 (50.0)	5 (50.0)	5 (50.0)		
30–39	5 (25.0)	3 (30.0)	2 (20.0)		
> 40	0 (0.0)	0 (0.0)	0 (0.0)		
Missing	2 (10.0)	0 (0.0)	2 (20.0)		
No. of ANC visits					
0	4 (20.0)	3 (30.0)	1 (10.0)		
1–2	1 (5.0)	0 (0.0)	1 (10.0)		
3–4	5 (25.0)	2 (20.0)	3 (30.0)		
> 4	7 (35.0)	4 (40.0)	2 (20.0)		
Missing	3 (15.0)	0 (0.0)	3 (30.0)		
No. of deliveries					
1–2	8 (40.0)	5 (50.0)	3 (30.0)		
3–4	3 (15.0)	1 (10.0)	2 (20.0)		
5–6	3 (15.0)	1 (10.0)	2 (20.0)		
> 6	5 (25.0)	3 (30.0)	2 (20.0)		
Missing	1 (5.0)	0 (0.0)	1 (10.0)		
Location of delivery					
Home	33 (82.5)	8 (80.0)	9 (90.0)	9 (90.0)	7 (70.0)
Health facility	6 (15.0)	2 (20.0)	0 (0.0)	1 (10.0)	3 (30.0)
Missing	1 (2.5)	0 (0.0)	1 (10.0)	0 (0.0)	0 (0.0)
Health outcome of newborn					
Died < 1 day of life	7 (35.0)			7 (35.0)	0 (0.0)
Died 1–7 days of life	2 (10.0)			2 (10.0)	0 (0.0)
Died 8–28 days of life	1 (5.0)			1 (5.0)	0 (0.0)
Survived 0–28 days of life	10 (50.0)			0 (0.0)	10 (100.0)
LGA					
Auyo	3 (7.5)	2 (20.0)	1 (10.0)	0 (0.0)	0 (0.0)
Miga	1 (2.5)	1 (10.0)	0 (0.0)	0 (0.0)	0 (0.0)
Kiyawa	4 (10.0)	2 (20.0)	0 (0.0)	0 (0.0)	2 (20.0)
Dutse	6 (15.0)	2 (20.0)	1 (10.0)	1 (10.0)	2 (20.0)
Gagarawa	1 (2.5)	1 (10.0)	0 (0.0)	0 (0.0)	0 (0.0)
Ringim	1 (2.5)	1 (10.0)	0 (0.0)	0 (0.0)	0 (0.0)
Yankwashi	1 (2.5)	1 (10.0)	0 (0.0)	0 (0.0)	0 (0.0)
Babura	1 (2.5)	0 (0.0)	1 (10.0)	0 (0.0)	0 (0.0)
Guri	2 (5.0)	0 (0.0)	1 (10.0)	0 (0.0)	1 (10.0)
Buji	7 (17.5)	0 (0.0)	6 (60.0)	0 (0.0)	1 (10.0)
Kafin Hausa	1 (2.5)	0 (0.0)	0 (0.0)	1 (10.0)	0 (0.0)
Gwiwa	2 (5.0)	0 (0.0)	0 (0.0)	2 (20.0)	0 (0.0)
Birninkudu	5 (12.5)	0 (0.0)	0 (0.0)	3 (30.0)	2 (20.0)
Kaugama	2 (5.0)	0 (0.0)	0 (0.0)	1 (10.0)	1 (10.0
Kirikasamma	2 (5.0)	0 (0.0)	0 (0.0)	2 (20.0)	0 (0.0)
Mallam Madori	1 (2.5)	0 (0.0)	0 (0.0)	0 (0.0)	1 (10.0)

The estimates represent the mean (SD) for continuous variables and frequency (%) for categorical variables

### Maternal death and reported PPH cases

#### Symptom recognition

Among maternal death cases, headaches were the most common symptom (seven cases), followed by fever (four cases), swelling (four cases), and bleeding (two cases). Unconsciousness, inability to talk or move and the presence of dafara—a thick, white saliva viewed as a sign of impending death—were each described in two cases. Other symptoms included vomiting (one case), paleness (one case), and shivering (one case).

Bleeding was reported in all PPH cases, while six cases described abdominal pain. Less common symptoms included headache (one case), dizziness (two cases), back pain (two cases), body pain (one case), vomiting (one case), and chest pain (one case).

Various actors were involved in recognizing symptoms in maternal death cases: the woman herself, her husband, other family members, and neighbors. In these cases, women were not always vocal about their symptoms and thus other household members were often crucial in symptom recognition, usually only once the condition had worsened. Female relatives and neighbors were the most common actors involved in symptom recognition for maternal death cases, though husbands were involved in three cases. In PPH cases, mothers-in-law, neighbors, and female relatives were involved, as were TBAs. Husbands, however, were not part of PPH recognition, likely since they are not traditionally present during the delivery process. “If a woman is in labor, he [the husband] can’t be there” (PPH-3). However, husbands continued to be key decision-makers in some cases.

Across maternal death and PPH cases, symptom recognition was driven by prior experience, knowledge, and perception of normalcy. For example, MD-4’s family member described how cold her body had become as she “had never felt such coldness on someone’s body before.” This signaled how unusual and shocking the symptoms were perceived as, and caused family members to lose hope. One respondent commented that “this had even made her lose hope on her survival.” Another woman described her views on a normal delivery: “Usually when a woman delivers, she smiles and speaks” (MD-2). A deviation from this pattern was seen as the cause for alarm. In another case, perceptions around normal and abnormal deliveries were also important: “…anyone that is going to give birth must discharge blood, even if a person delivers in a hospital” (PPH-5). Perceptions of bleeding as a problematic or non-problematic symptom were often influenced by experiences during past deliveries. For example, one respondent stated “whenever she gives birth, she usually encounters bleeding” (PPH-9).

Assessment of symptom severity hinged on sudden, disruptive changes in the woman’s well-being and her ability to undertake her usual activities. One relative stated “the headache was not that serious, since she was talking and drinking water with her family” (MD-1). Another family noted that “it was very serious indeed, at the time we were going to the hospital, she wasn’t able to enter into the car by herself, until she was lifted” (MD-8). Some symptoms such as dafara were ascribed the highest severity level: “…dafara is saliva with bitter taste, if you see it accumulating in a sick person’s mouth, then you should know that he will not survive” (MD-2). Bleeding severity was determined by quantity and flow of blood, with heavy flow considered severe. “I bled so much, almost all the wrappers in my drawer were soaked with blood” (PPH-2). Another family member explained “If the flow of blood is heavy, then it means there is problem, and it can be easily identified” (PPH-8). The presence of additional symptoms beyond bleeding such as shivering, unconsciousness, paleness, and weakness was associated with higher severity. For example, a family member describes the woman’s bleeding as not severe at first, but then “it was [severe] the following day around the late afternoon, when her body became weak” (PPH-6).

Medical causes described in maternal death cases include retained placenta, “inadequate blood”—the local phrase for anemia, and eclampsia, while spiritual causes included attack by spirits (one case) or the will of God (four cases). For the most part, medical and spiritual causes were mutually exclusive. Families reported either medical or spiritual causes but not both. In addition, there were a number of cases where family members expressed that they did not know the cause of the symptoms (four cases) but, when pressed, attributed them to God. Headache, the most frequently mentioned symptom in maternal death cases, was seen as a normal sign of impending delivery among families of women who died. In PPH cases, most women attributed bleeding to the delivery process (six cases), one to excess blood sugar, and three to God. Overall, God was mentioned in seven maternal death and five PPH cases as an explanation or contributor to the illness and/or death. God was also described as the ultimate controller of outcomes, highlighting the fatalism and the perceived inability to control final consequences that many respondents expressed. “It is fate. If God gives you safe delivery, you are fortunate enough, and if you didn’t get safe delivery, then the result is just like this one” (MD-2).

#### Decision-making

For maternal death cases, the husband was the key decision-maker with respect to care-seeking. When husbands were absent, relatives including the husband’s brothers, sisters, or mother (two cases) and/or neighbors (two cases) jointly made decisions about how to handle the symptoms, including whether or not to seek care and what type of care to seek and how. In PPH cases, the husband was the decision-maker in four cases but almost always made decisions together with a co-wife. In most of the remaining cases, female relatives made the key decisions. In two cases, TBAs were present and made the decision to seek care at a health facility.

In both cases where TBAs were present, they encouraged immediate health facility care and, in one case, strongly discouraged the use of traditional medicine. The evidence suggests TBAs have significant authority within the household, as families trust them and implement their decisions immediately. Families also feel at ease because TBAs are able to navigate the health care system. One TBA stated: “They (the family) are the ones that called me…I am the one that takes pregnant women to the hospital, I am familiar with the health workers, they are my friends” (PPH-4).

In addition to the presence of TBAs, decision-making was also enabled by the recognition of the severity of symptoms, an understanding of the causes of symptoms, proximity to a health facility, positive attitudes about care-seeking, and the ability to mobilize resources and transportation. One woman explained that purchasing drugs from a chemist is usually the first line of action, unless the symptoms are considered to be very severe: “whenever any problem of sickness arises, we buy drugs, or we just go to hospital if the case is a major one” (PPH-7). The family of MD-4, who had facial swelling, followed by fever and difficulty breathing, described how the absence of understanding about the cause of the problem was a barrier to decision-making: “Honestly, we did not do anything, because we didn’t know what was wrong with her” (MD-4). However, once symptoms perceived as being very severe such as dafara appeared, the family made the decision to go to the hospital.

Other barriers included the absence of health workers, and fear of health workers, or fake drugs. Lack of money or available transportation was the most commonly cited barrier and often contributed to the death. MD-6, who suffered first from headache and swollen face and body while pregnant, was given traditional medicines for these symptoms. However, once her illness progressed and she became unconscious after delivering at home, the family decided to take her to the hospital but was unable to find transportation: “All in all she was not taken away from home, she died while we were trying or in the process of looking for car” (MD-6). In the case of MD-5, who suffered from kidney problems, monetary resources could not be mobilized in time to pay the doctor, despite the family’s best efforts. The deceased’s sister recounts that “It was N37,000 [for her treatment] and altogether N50,000 including bed fees [admission fees], but she died before the time. On hearing this amount of money, we all gave up.”

#### Care-seeking

**Fig. 1:**
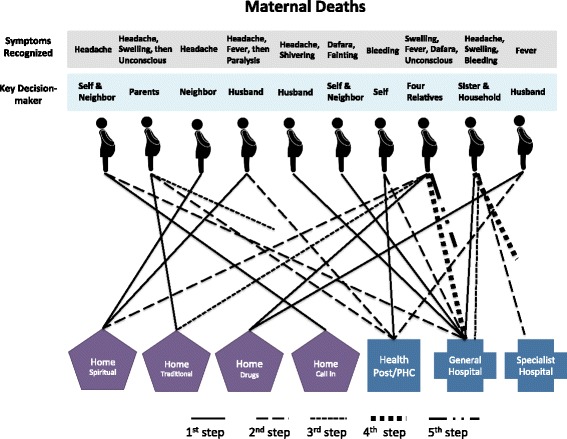
Care-seeking steps taken in maternal death cases

**Fig. 2:**
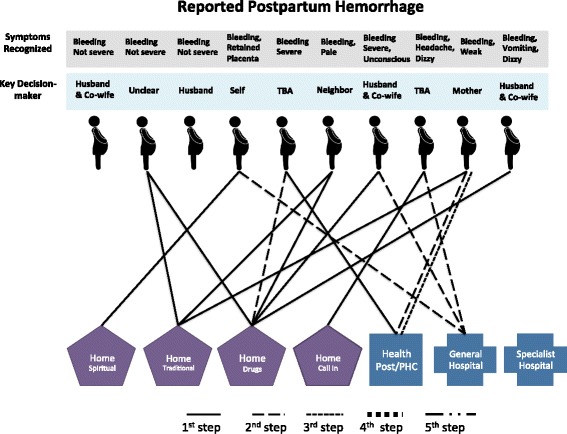
Care-seeking steps taken in reported postpartum hemorrhage cases

Once the decision was made, families sought care for the illnesses through a variety of channels, including home care via drugs, traditional medicine (such as herbs or plants) or spiritual remedies (such as prayers and recitations), as well as home visits by health care providers and facility-based care. The pattern and sequence of care-seeking for the ten deceased women are illustrated in Fig. 1 and those for the ten PPH cases in Fig. 2. In these figures, each case is represented by an icon of a pregnant woman and each step that is taken to obtain care for the focal woman is indicated by a solid or dotted line, leading to a specific type of home-based or facility-based care. The specific symptoms recognized for each case and the key decision-maker are listed above each icon to facilitate comparison across individual cases.

As can be seen in Fig. 1, in six of the maternal death cases, the first care-seeking step (which is represented by the solid lines) involved home care. For example, in the first case (represented by the first icon in Fig. 1), the woman experienced headache and she and her neighbor made a decision to call a local health worker to their home to examine her. He promised to return the next morning, but her condition did not improve and they decided to, as a second step (represented by the dashed line), visit the general hospital.

In three cases, spiritual care such as tofi, an act where Koranic verses are recited and air is blown over painful body parts, was sought, often as a first step. All three of these cases were perceived as being very serious, and two cases included symptoms such as paralysis, dafara, and unconsciousness. The two cases that utilized traditional medicines were also described as being very serious and included unconsciousness, and in one case, dafara. All maternal death cases sought some type of care within a day of recognition, and all but one visited a health facility at some point. Once facility-based care was sought, subsequent referrals were common. Three cases (MD-4, MD-5, MD-6) died either en route to or while securing resources to visit a second facility. The sequence of steps involved during care-seeking seemed to depend on the perceived severity and cause as well as the location of the treatment that is perceived to address the problem. One husband explained why family members went to buy drugs from a chemist as the first step in seeking care for his wife who had a fever: “We went there because it was close to us, whenever we got a minor problem like stomach pain or fever we go there, until the problem is beyond there before we go to the hospital” (MD-8). Another respondent felt that “the best advice is to go where one can get remedy, so the best thing is to go to the hospital” (MD-1).

In PPH cases (Fig. 2), the first step was home care in seven cases, facility-based care in one case, and no action in two cases. In both cases where no care was sought, the bleeding was not perceived as being serious. Five cases eventually sought care at a health facility. All of these were considered to have severe bleeding by family members, and most had accompanying symptoms such as paleness, weakness, and loss of consciousness. There were, on average, fewer steps per case for PPH cases (1.5) compared to maternal death cases (2.1).

**Fig. 3:**
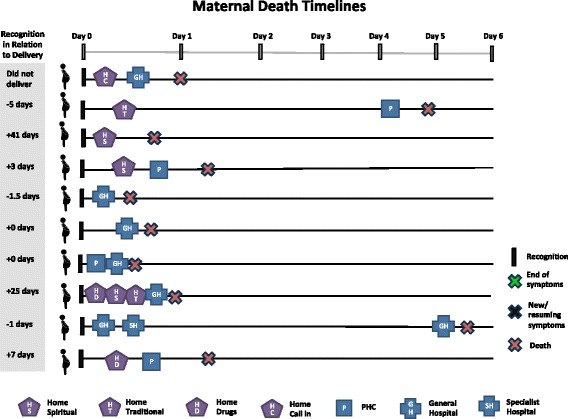
Timing and location of care-seeking in maternal death case

**Fig. 4:**
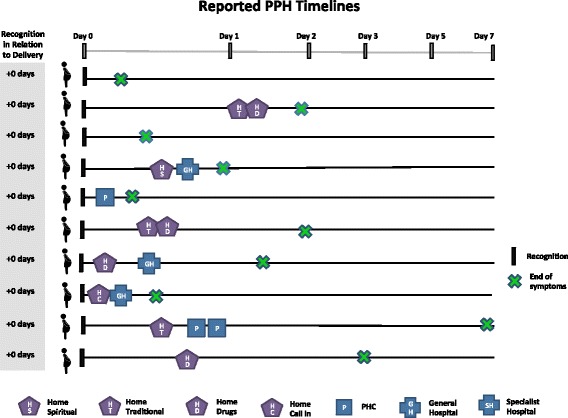
Timing and location of care-seeking in reported postpartum hemorrhage case

Care-seeking timelines for maternal death and PPH cases are shown in Figs. 3 and 4, respectively. Day zero is the point of symptom recognition. Care-seeking began very shortly after symptom recognition for most maternal deaths. Six deaths occurred within 24 h of recognition, and eight occurred within 48 h. Across PPH cases, there is a variation in the time between symptom recognition and the first care-seeking step and also a delay in care-seeking compared to maternal deaths. In some PPH cases, this time was influenced by disagreements in which step to take: “it wasn’t more than an hour, because some were saying more concoction [traditional medicine] should be given to her, while others were saying no, she should just be taken to the hospital” (PPH-8). In other cases, the delay was related to perceptions of normality and inability to recognize severity, while in others, it was due to changes in symptoms and other barriers such as lack of money. Symptoms were resolved in eight of the PPH cases within 48 h after symptom recognition.

### Newborn death and illness cases

#### Symptom recognition

Among the ten neonatal death cases, paleness of the body and eyes was the most common symptom (six cases). One woman described her newborn’s symptoms: “She was pale and it was as if she had no blood” (ND-3). Another mother stated that her baby “was opening his eyes widely and they had turned greenish, and he also became pale” (ND-1). Other reported symptoms include low body temperature (three cases), difficulty breathing (two cases), excessive crying (two cases), discharging whitish saliva (two cases), inability to cry (one case), weight loss (one case), constipation (one case), bleeding from mouth and nose (one case), convulsion (one case), and fever (one case). In two cases, families stated that the newborns were born early or before it was time. In almost all neonatal death cases, symptoms were first recognized by the mother. The mother’s mother or co-wives were also involved in the recognition process, but husbands were never involved.

Excessive crying was reported in six neonatal illness cases and fever in five cases. Other symptoms include rashes (three cases), vomiting (three cases), swollen stomach (two cases), cough (one case), stomach pain (one case), ear ache (one case), and breast pain and swelling (one case). These symptoms spanned longer periods and were often more specific than in newborn death cases. Symptoms were first recognized by the mother, who then called for her husband’s opinion.

Perceptions of severity were influenced by previous experience and beliefs about abnormal behavior. For example, one mother described her baby’s abnormal crying: “…I started thinking that maybe she was sick, because I don’t usually see babies crying the way she has been crying” (ND-10). Parents looked for visible signs that were deviations from normality. For example, one mother explained: “This problem is easily identified, because you will see that the child’s stomach will be swelling, especially if he is vomiting” (NI-5). Severity was also judged by symptom magnitude and how widespread they were. For example, one family member said: “Well, it was very serious since the rashes appeared all over her body” (NI-1). A symptom that was short-lived was not considered to be serious: “The cough wasn’t so serious, because it did not last for long” (NI-1). Being born early was considered to be a very severe problem that significantly reduces the chances of survival. One woman stated about a woman who delivered when she was 7 months pregnant: “The reason why I became frightened was because the baby has been delivered before it was time… she [the baby] didn’t survive” (ND-7).

Perceived causes in neonatal death cases ranged from illnesses described as yellow fever in three cases, to jaundice, to common cold. Several symptoms were attributed to an illness described as “ta yara” or *yellow fever* including body pallor and discharge of saliva: “If you see saliva coming out from a baby’s mouth at the time of delivery, then know that it is ‘ta yara’” (ND-7). Green eyes were described by one family as being caused by an illness they referred to as *jaundice* and best treated with traditional medicines.

In newborn illness cases, perceived causes ranged from malaria to common cold to flu. In the case of malaria, the English word “malaria” was used to describe a fever caused by a mosquito bite. In two cases, frequent vomiting and milk regurgitation were considered as normal events. The will of God was mentioned as a contributor to the problem in seven newborn death and four newborn illness cases.

#### Decision-making

In neonatal death cases, decision-making was primarily undertaken by the newborn’s mother or the mother’s mother. In two cases, the decision was made by the husband, while in one case, it was made by a health worker called into the home. In contrast, the baby’s father was a key decision-maker in neonatal illness cases (eight cases).

Factors that enabled decision-making, including early symptom recognition, household consensus around appropriate actions, and resource availability, were similar to maternal death and PPH cases. In neonatal death cases, rapid onset of symptoms and progression of the disease were identified several times as a barrier to care-seeking. One mother explained: “We didn’t make any attempt [to seek care], because she died shortly after delivery” (ND-8). A family member of a different newborn who died stated: “As the flow of blood was heavy everyone agreed that nothing should be done to her at home, but should be taken to the hospital, both the paternal and maternal sides agreed upon. But before taking any further step, she died, we were really surprised about her death, it was very fast” (ND-4).

Other obstacles included lack of resources and an inability to access care at the point of symptom onset at night, when health facilities are closed or unstaffed. One father explained how perception of severity influenced the decision not to seek care: “Concerning the vomiting, nothing came into my mind, because I did not consider it to be a problem… I did not take it to be an illness, so I was thinking that it will stop.” The mother concurred: “We thought that it wasn’t an illness that was why we did not take him for treatment” (NI-2). Timing of symptom onset was also described as a barrier: “You know it all happened at night, so nothing can possibly be done before morning, since we don’t have a hospital here, had it been there is a hospital here, then she would have been taken to the hospital immediately, because her brother has got a vehicle, so he can convey them. Therefore nothing has been done for the baby” (ND-7). Belief in traditional medicine and perceptions on prematurity also impeded decisions to seek care: “We honestly didn’t think of doing anything because she was a premature baby” (ND-8).

#### Care-seeking

**Fig. 5:**
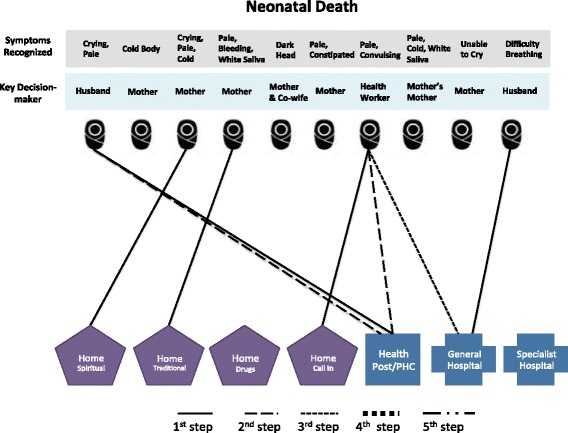
Care-seeking steps taken in neonatal death cases

**Fig. 6:**
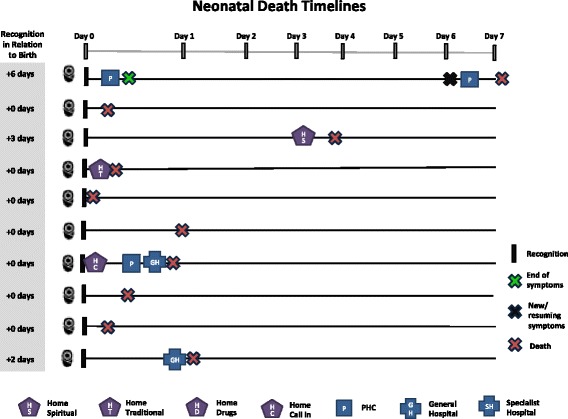
Timing and location of care-seeking in neonatal death cases

Compared to maternal cases, little care was sought for newborn death cases (Fig. 5). In five of the ten cases, no care of any type (home- or facility-based) was sought. In most of these cases, the mother was the key decision-maker and the death occurred very quickly before action could be taken. In one of the cases where no care was sought, symptoms appeared at night when the health facility was closed, leading to a decision to wait until morning. The baby, however, died before morning. In four of the neonatal death cases, home care, usually traditional medicine, was the first step, followed by a health facility visit if symptoms did not improve. Overall, only three cases eventually sought care at a health facility. Lack of care-seeking seems to be linked to the rapid death of many of the newborns—five out of ten died within hours, and seven out of ten died within 1 day of birth (Fig. 6). The average number of care-seeking steps for neonatal death cases was 0.9 compared with 2.1 steps per maternal death case.

**Fig. 7:**
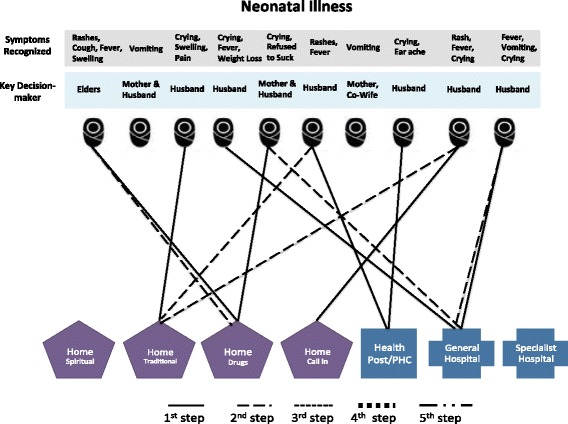
Care-seeking steps taken in neonatal illness cases

**Fig. 8:**
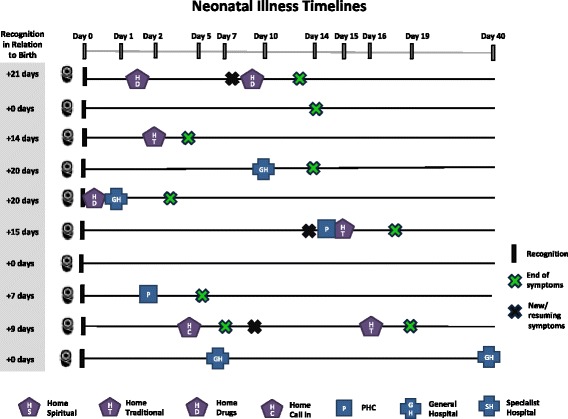
Timing and location of care-seeking in neonatal illness cases

In two newborn illness cases, no care was sought. In both cases, the decision-maker was the mother and the baby’s main symptom was vomiting which was not believed to be serious (Fig. 7). In eight cases, care was sought, with half seeking care at home and half at a facility as the first step. Overall, five cases eventually sought care at a health facility. The average number of care-seeking steps for neonatal illness cases was 1.3. This is higher than neonatal deaths (0.9 step) but comparable with perceived PPH cases (1.5 step). Figure 8 shows substantial variations among neonatal illness cases in the time between symptom recognition and the first step of care and in the time to illness resolution.

### Enablers and barriers of care-seeking at health facilities

Enablers of care-seeking at health facilities across all case types included strong belief in and trust of the medical system, proximity to health facilities, readily available transport and funds, and family member support to seek medical care. Many barriers were also identified. Securing resources such as money and transport was a common challenge. In some cases, the nearest medical facility was not open when needed, or no health worker was present. These barriers strongly influenced the decision-making process. For example, in one neonatal death case described above, the symptoms began during the night when the health facility was closed. Despite wanting to seek care immediately, the family decided to wait until the morning when the facility would be open. The baby died before care could be sought.

In the maternal death cases, especially, health system challenges were critical in the death. One family described attempts to obtain help at the facility:

*“We decided to go to Hadejia because the doctor we first met did not do anything since around 7am. We went to pick another doctor whom later we realised that we would not get genuine drugs from him, so we went to the hospital (5:15pm). She was given admission (5:20pm) but nothing was done till the following morning, which they asked us to buy some drugs (5:30am). First, we were asked to buy hand gloves, then some drugs, then second batch of drugs and later asked to buy materials for admission. After buying those things and I sat for a few moments, then I was informed she has passed away”* (MD-1).

Ultimately, for the maternal death cases, more time was spent waiting at the facilities to receive care than was spent in securing resources or in traveling to the facilities. The third delay in Thaddeus and Maine’s three-delay model seemed to be the most lengthy and critical delay. This contrasts with PPH cases where long delays at health facilities were not experienced. For example, in one PPH case, one respondent explained: “Immediately on reaching the hospital, when they [health workers] heard her problem, they attended to her….they attend to us quickly if it is a case of bleeding, even if it is at night” (PPH-4).

The most significant barrier for newborn death cases appeared to be rapid illness progression to death, leaving inadequate time to seek care. Belief in traditional medicines for illnesses such as yellow fever also delayed or prevented care-seeking at a health facility. In one case, the mother explained: “I made the decision to have the rubutu [a traditional medicine] prepared, because I thought she would feel better…In my opinion, it was helpful to her since she stopped crying” (ND-3). However, this perceived improvement prevented any further action, and the baby died.

### Risk perception

Almost universally, males and females described high risks associated with pregnancy. One woman stated: “Because of its hardship, immediately when you become pregnant, you are being regarded as someone near to death, even you are counting yourself among the dead, until the day you deliver safely” (ND-7). The view that pregnant women are straddling life and death was expressed by several respondents, and in a Hausa proverb: “From the time when a woman becomes pregnant… her life is at risk. According to the Hausa people ‘one leg of a pregnant woman is in the world, while the other one is in the heaven’” (MD-9).

Respondents also consistently expressed the belief that pregnancy outcomes are ultimately determined by God. However, despite this belief, many actions were still taken to cure illness. In maternal death cases especially, spiritual care was sought via religious figures or family members. At times, this delayed care-seeking at a facility and contributed to eventual death.

## Discussion

The study findings describe patterns of recognition, decision-making, and care-seeking among families of women who died during pregnancy, delivery, or postpartum or who experienced perceived PPH, and newborns who died or experienced an illness during the neonatal period. The findings shed light on the differences and similarities by case type as summarized in Table 3, and how these contribute to the three delays described in Thaddeus and Maine’s three-delay model. Compared to maternal cases, less care-seeking was reported for newborns, especially in cases that ended in death. This lack of care-seeking for newborns was driven by greater challenges in timely recognition of symptoms and their severity, often due to their non-specificity, as well as rapid progression of illness to death. This finding is consistent with several other studies [14, 15]. Similar to other research, this study found that perceptions of severity and attributed cause are important influencers of care-seeking for both maternal and newborn cases [16, 17]. Challenges in recognition of the illness and its severity contribute to the first delay in the three-delay model, along with delays due to decision-making. Delays due to recognition seemed to be most pronounced in maternal and newborn death cases.

**Table 3:** Recognition, decision-making, and care-seeking by case type

Case type	Recognition	Decision-making	Care-seeking
Maternal death	Variable depending on symptom; often only when symptoms are very severe and woman’s daily functioning is affected Female relatives, neighbors, husbands (3 cases)	Husbands, other family members (husband’s brothers, sisters, or mother), or neighbors if husband is absent	9 of 10 cases went to health facility; 8 of the 9 within 24 h Spiritual care (prayers and tofi) used in 3 of 10 cases, contributed to delays in care-seeking at facility 6 of 9 cases faced significant barriers at the health facility level
Perceived PPH	Quick (blood is very obvious) Female relatives, TBAs; no husbands were involved	Female relatives, TBAs; only a few husbands (when involved usually together with co-wives)	5 of 10 cases went to a health facility Fewer barriers at health facility (no long waits)
Neonatal death	Variable; some symptoms such as paleness and constipation were not considered to be severe Mother; sometimes mother’s mother or co-wives were also involved. Husbands were never involved	Mostly the mother or mother’s mother; only a few husbands; health worker in one case	3 of 10 cases went to a health facility Symptom progression and death occur too quickly for care-seeking Traditional medicine impeded care-seeking 2 of 3 cases faced some barriers at the health facility level (long waits and multiple referrals)
Neonatal illness	Variable; quicker when symptoms are specific and visible Mother first; she then called for husband’s opinion	Husbands (in 8 of 10 cases)	5 of 10 cases went to a health facility Longer delays between symptom recognition and care-seeking Few delays at the health facility level

Key decision-makers varied by case type, with husbands playing the lead role in maternal death and neonatal illness cases, while female relatives and TBAs were more involved in decision-making around perceived PPH, and mothers were the principal decision-makers in the neonatal death cases. This finding was somewhat surprising, as males are often reported as key health decision-makers in Nigeria. For example, in Kaduna, Northern Nigeria, 63% of women required permission from their husbands to visit a hospital [18]. Our results are more nuanced, suggesting that decision-makers vary depending on type and severity of the illness, and whether the mother or newborn is affected. For example, female relatives were a key in decisions related to PPH, while husbands and male relatives were more involved when other maternal illnesses involving headache, weakness, and fever were present. The relative negotiating power of women versus men in these families may have contributed to the observed differences in decision-making, but this could not be assessed with the available data.

In cases where there was disagreement about appropriate action to be taken, or uncertainty about the cause, the slower decision-making process led to increases in delay 1. However, these decision-making delays were not particularly linked to a specific case type.

Strong trust in the health care system and willingness to seek care were consistent across case types but were impeded by barriers such as cost, distance to health facilities, availability of health workers, and drug stock-outs. These obstacles contribute to both delays 1 and 2 and are consistent with other findings in Northern Nigeria [8, 10, 11]. For example, barriers such as lack of money and transportation influenced the decision-making process, thus contributing to delay 1, but in addition, once a decision was made to seek care, these obstacles also led to delays in reaching the health facility (delay 2). Moreover, health system challenges, such as availability of health care providers, also contribute to delay 3. These were critical factors in the maternal death cases and, to a lesser degree, the neonatal death cases but seemed less important for other case types. For example, while many maternal death cases experienced long delays at facilities and some died while waiting, none of the reported PPH cases who sought care at a facility reported delays in obtaining care. Since only two of the maternal deaths involved bleeding, further investigation of the link between maternal complication type and delay at health facilities is warranted.

The largest barrier in the newborn death cases seemed to be rapid progression of symptoms from the moment of recognition to death, leaving no time for care utilization. This was linked to poor timely recognition and influenced by the presence of non-specific symptoms. The third delay in newborn death cases is difficult to assess, as few cases reached a facility prior to the infant’s death. However, of the three newborn death cases that reached a health facility, two faced substantial delays at the facility.

Finally, childbearing is seen as a dangerous event by both men and women, but most families believe actions can and should be taken even if outcomes are ultimately controlled by God. These actions include attending antenatal care, seeking care at a health facility when there is a serious complication, or utilizing care at home when the illness is less severe. This belief in God delayed care-seeking at a health facility in several of the maternal death cases when spiritual care was utilized, but was not a major factor in other case types.

In Sub-Saharan Africa, several other studies also identified fatalistic views regarding maternal complications [19–21] and newborn survival [22] and the use of spiritual care to influence God [19, 23, 24]. In Ethiopia, respondents expressed the belief that maternal complications were controlled by God’s will, and described attempts to influence God through prayer and the resulting delays in other care-seeking actions [19]. The importance of spiritual care was also described in South West Nigeria, where 75.8% of women interviewed expressed a need for spiritual help during pregnancy and childbirth, and 70.8% believed that health care providers should consider their spiritual needs [23]. Another study in Kano, Nigeria, found that almost 30% of women believed that eclampsia could be best managed through spiritual means [24]. In our study, fatalism was not provided as a reason to avoid seeking care, but rather as an explanation for adverse outcomes. However, in cases where it was believed that spiritual care such as tofi and prayers could influence God’s will, delays in seeking other types of care occurred.

In addition, belief in and the use of traditional medicine such as rubutu were also widespread and there was delayed care-seeking at health facilities for both mothers and newborns. This is consistent with other findings in Nigeria which underscore the very high levels of acceptance and the use of traditional medicine among pregnant women [25, 26] and in neonates [27]. Our study finds that delays in care-seeking related to the use of traditional medicines were most pronounced in neonatal cases and, in at least one case, directly contributed to the eventual death of a newborn.

This study has several strengths. First, it includes a sample drawn from 24 distinct LGAs. The study drew its sample from a large cohort of women being followed prospectively and involved case-finding using real-time surveillance. These elements increase diversity of the sample and ensure the inclusion of perspectives from a wide geographic area. The inclusion of both maternal and newborn cases, as well as cases resulting in death and those where the illness was resolved, allows for novel comparisons. Finally, the creation of detailed timelines allowed systematic comparison of the sequence and timing of care-seeking.

Limitations include the use of self-reported data that may be subject to recall bias. To minimize this bias, the majority of cases were visited within weeks of the death or illness. Social desirability bias could have influenced responses, but interviewers were extensively trained in strategies to build trust and rapport with respondents. In addition, as with all qualitative research, external validity is limited. Finally, clinical data to validate reported symptoms was largely absent. While such data would have been useful, the objective was to understand recognition and care-seeking from the perspective of the families.

Our findings have implications for future health programming, policy, and research. Interventions developed for Northern Nigeria should carefully tailor educational messaging and other strategies to case type. Interventions targeting maternal care-seeking, for example, require different messages than those focused on newborn care-seeking. While bleeding was generally recognized as an urgent maternal complication, clearer messages on other symptoms as well as where to obtain appropriate care would be helpful, as would the implementation of community-based schemes to ensure transportation and access to financial resources, especially at night. For newborns, messages on danger signs, especially on their non-specificity, and the importance of seeking immediate care are needed as illnesses can progress rapidly to death.

Demand for health services is high, but supply-side challenges such as quality of care and availability of health workers and drugs continue to be a problem despite the implementation of the MSS and must be urgently addressed. Maternal and newborn interventions that do not already address the barriers posed by perceived spiritual causes of illness and the importance of spiritual care should also be sought to address this barrier in their programming. Finally, key decision-makers should be targeted, but this must go beyond including husbands and also involve both male and female relatives who are instrumental in decision-making.

## Conclusion

These findings suggest key differences in recognition of complications, decision-making processes, and care-seeking patterns between maternal and newborn cases in Jigawa, Northern Nigeria. Less care is sought for newborns than for maternal complications often due to symptom non-specificity and rapid progression of illness to death, and the key decision-maker with respect to care-seeking varies depending on the type of case, timing of the symptoms, and perceived severity. Health systems are weak and contribute to poor health outcomes, especially for women with maternal complications. Public health interventions that provide more targeted messaging by case type, are inclusive of family members beyond husbands, and address gaps in quality and availability of care are urgently needed. Fatalistic views related to maternal and newborn outcomes are prevalent and should also be addressed by interventions.

## Abbreviations

CoRPs: Community resource persons
J-PAL: Abdul Latif Jameel Poverty Action Lab
LGA: Local government area
MIT: Massachusetts Institute of Technology
MMR: Maternal mortality ratio
MSS: Midwives Service Scheme
NMR: Neonatal mortality rate
ORAC: Operations Research Advisory Committee
PHC: Primary health center
PPFN: Planned Parenthood Federation of Nigeria
PPH: Postpartum hemorrhage
RCT: Randomized controlled trial
SBA: Skilled birth attendant
TBA: Traditional birth attendant
